# Clinical practice guidelines for the diagnosis and surveillance of *BAP1* tumour predisposition syndrome

**DOI:** 10.1038/s41431-023-01448-z

**Published:** 2023-08-22

**Authors:** Fiona Lalloo, Anju Kulkarni, Cindy Chau, Maartje Nielsen, Michael Sheaff, Jeremy Steele, Remco van Doorn, Karin Wadt, Monica Hamill, Beth Torr, Marc Tischkowitz, Delphi respondents, Munaza Ahmed, Svetlana Bajalica-Lagercrantz, Ana Blatnik, Joan Brunet, Ruth Cleaver, Chrystelle Colas, Tabib Dabir, D. Gareth Evans, Shirin Feshtali, Paola Ghiorzo, Lise Graversen, Klaus Griewank, Hildur Helgadottir, Rosalyn Jewell, Kelly Kohut, Henrik Lorentzen, Daniela Massi, Guy Missotten, Alex Murray, Jennie Murray, Ernest Nadal, Kai Ren Ong, Josep M. Piulats, Susana Puig, Neil Rajan, Simone Ribero, Galateau Salle, Alexandre Teulé, Emma Tham, Barbara van Paassen, Robin De Putter, Robert Verdijk, Anja Wagner, Emma R. Woodward, Helen Hanson

**Affiliations:** 1 Manchester Centre for Genomic Medicine, Manchester University NHS Foundation Trust, Manchester, UK; 2 Department of Clinical Genetics, Guy’s and St Thomas’ NHS Foundation Trust, London, UK; 3 Department of Ophthalmology, Leiden University Medical Center, Leiden, the Netherlands; 4 Department of Clinical Genetics, Leiden University Medical Center, Leiden, the Netherlands; 5 Department of Cellular Pathology, Barts Health NHS Trust, London, UK; 6 Department of Oncology, Barts Health NHS Trust, London, UK; 7 Department of Dermatology, Leiden University Medical Center, Leiden, the Netherlands; 8 Department of Clinical Genetics, Copenhagen University Hospital, Copenhagen, Denmark; 9 Division of Genetics and Epidemiology, Institute of Cancer Research, Sutton, London, UK; 10 Department of Medical Genetics, National Institute for Health Research Cambridge Biomedical Research Centre, University of Cambridge, Cambridge, UK; 11 South West Thames Regional Genetics Service, St George’s University Hospitals NHS Foundation Trust, London, UK; 12 North East Thames Regional Clinical Genetics Service, Great Ormond Street Hospital for Children NHS Foundation Trust, London, UK; 13 Centre for Rare Diseases, Karolinska Universitetssjukhuset, Stockholm, Sweden; 14 Department of Clinical Cancer Genetics, Institute of Oncology Ljubljana, Ljubljana, Slovenia; 15 Hereditary Cancer Programme, Catalan Institute of Oncology, IDIBGI, Girona, Spain; 16 Peninsula Genetics Service, Royal Devon University Healthcare NHS Foundation Trust, Exeter, UK; 17 Department of Genetics, Institut Curie, INSERM U830, Université de Paris -Cité, Paris, France; 18 Northern Ireland Regional Genetics Centre, Belfast City Hospital, Belfast, UK; 19 Division of Evolution and Genomic Science, The University of Manchester School of Health Sciences, Manchester, UK; 20 Department of Radiology, Leiden University Medical Centre, Leiden, the Netherlands; 21 Dipartimento di Medicina Interna, Università degli Studi di Genova, Genoa, Italy; 22 Genetics of Rare Cancers, IRCCS Ospedale Policlinico San Martino, Genoa, Italy; 23 Department of Clinical Genetics, Aarhus University Hospital, Aarhus, Denmark; 24 Dermatology Department, University Hospital Essen, Essen, Germany; 25 Department for Oncology-Pathology, Karolinska Institutet, Stockholm, Sweden; 26 Yorkshire Regional Genetics Service, Leeds Teaching Hospitals NHS Trust, Leeds, UK; 27 Department of Clinical Genetics, St George’s University Hospitals NHS Foundation Trust, London, UK; 28 Department of Dermatology, Aarhus University, Aarhus, Denmark; 29 Section of Pathology, Department of Health Sciences, University of Florence, Florence, Italy; 30 Department of Ocular Oncology, Leuven University Hospitals, Leuven, Belgium; 31 All Wales Medical Genomics Service, Cardiff, UK; 32 South East Scotland Clinical Genetics Service, Western General Hospital, Edinburgh, UK; 33 Department of Medical Oncology, Catalan Institute of Oncology, Bellvitge Biomedical Research Institute, L’Hospitalet, Spain; 34 West Midlands Regional Genetics Service, Birmingham Women’s Hospital, Birmingham, UK; 35 Department of Medical Oncologist, Institut Català d’Oncologia, L’Hospitalet de Llobregat, Spain; 36 Servicio de Dermatología, Hospital Clínic de Barcelona, Barcelona, Catalonia Spain; 37 Translational and Clinical Research Institute, Newcastle University, Newcastle upon Tyne, UK; 38 Dermatology clinic, Department of medical sciences, University of Torino, Torino, Italy; 39 Department of Pathology, Centre Hospitalier Universitaire de Caen, Caen, France; 40 Hereditary Cancer Program at Catalan Institute of Oncology, l’Hospitalet de Llobregat, Catalonia Spain; 41 Department of Molecular Medicine and Surgery, Karolinska Institutet, Stockholm, Sweden; 42 Clinical Genetics, Karolinska University Hospital, Stockholm, Sweden; 43 Department of Clinical Genetics, Erasmus MC, Rotterdam, the Netherlands; 44 Center for Medical Genetics, Ghent University and Ghent University Hospital, Ghent, Belgium; 45 Manchester Centre for Genomic Medicine, MFT, Manchester, UK; 46 University of Manchester, Manchester, UK

**Keywords:** Cancer genetics, Cancer genetics

## Abstract

BRCA1-associated protein-1 (BAP1) is a recognised tumour suppressor gene. Germline *BAP1* pathogenic/likely pathogenic variants are associated with predisposition to multiple tumours, including uveal melanoma, malignant pleural and peritoneal mesothelioma, renal cell carcinoma and specific non-malignant neoplasms of the skin, as part of the autosomal dominant *BAP1*-tumour predisposition syndrome. The overall lifetime risk for *BAP1* carriers to develop at least one *BAP1*-associated tumour is up to 85%, although due to ascertainment bias, current estimates of risk are likely to be overestimated. As for many rare cancer predisposition syndromes, there is limited scientific evidence to support the utility of surveillance and, therefore, management recommendations for *BAP1* carriers are based on expert opinion. To date, European recommendations for *BAP1* carriers have not been published but are necessary due to the emerging phenotype of this recently described syndrome and increased identification of *BAP1* carriers via large gene panels or tumour sequencing. To address this, the Clinical Guideline Working Group of the CanGene-CanVar project in the United Kingdom invited European collaborators to collaborate to develop guidelines to harmonize surveillance programmes within Europe. Recommendations with respect to *BAP1* testing and surveillance were achieved following literature review and Delphi survey completed by a core group and an extended expert group of 34 European specialists including Geneticists, Ophthalmologists, Oncologists, Dermatologists and Pathologists. It is recognised that these largely evidence-based but pragmatic recommendations will evolve over time as further data from research collaborations informs the phenotypic spectrum and surveillance outcomes.

## Introduction

BRCA1-associated protein-1 (*BAP1*) was identified as a tumour suppressor gene in 2008 [1]. Germline pathogenic variants (GPV, including likely pathogenic variants) in *BAP1* have subsequently been associated with a variety of tumours resulting in the recognition of *BAP1*-associated tumour predisposition syndrome (*BAP1*-TPDS) [2, 3]. GPV were originally associated with familial occurrence of cutaneous melanocytic tumours but the spectrum of tumours is now accepted to include uveal melanoma, malignant mesothelioma of the pleura and the peritoneum, renal cell carcinoma and specific non-malignant neoplasms of the skin. More recently additional tumour types including meningioma, cholangiocarcinoma and hepatocellular carcinoma have been suggested as possible associations with *BAP1*-TPDS [2, 4].

Lifetime cancer risks for individuals with a *BAP1* GPV (henceforth referred to as *BAP1* carriers) are reported to be 20–25% for mesothelioma, uveal and cutaneous melanoma, with lower lifetime risks reported for renal cell carcinoma and other associated cancers. The overall lifetime risk for at least one *BAP1*-associated tumour is up to 85% [2, 5]. However, due to ascertainment bias, current estimates of cancer risk are likely to be overestimated.

*BAP1* GPV are inherited in an autosomal dominant manner and once a GPV is identified in an individual, predictive (also known as pre-symptomatic) testing can be offered to relatives. This facilitates assessment of cancer risk and discussion of options for early detection and/or cancer prevention. De novo GPV in *BAP1* have been reported in almost 10% (2/21) in a Dutch patient cohort [4]. The exact incidence is unknown but considered low.

For almost all rare cancer predisposition syndromes there is limited scientific evidence of reduced morbidity or mortality for surveillance, meaning it is difficult to achieve the fine balance between too much surveillance (leading to unnecessary further investigations such as biopsy), and too little (resulting in missed opportunities to detect a cancer at a treatable stage). Opinions on surveillance can vary greatly between clinicians based on experience and the health care systems in which they work.

Whilst surveillance guidelines have been published from American and Australian groups [5, 6] (Supplementary Table 1) there remains limited evidence and conflicting recommendations. To date, European recommendations have not been published. In a survey of UK Genetics centres in late 2019, *BAP1* was identified as a gene considered to be a priority for guideline development (personal communication H. Hanson). Guidelines are necessary due to both the emerging phenotype of this recently described syndrome and increased identification of *BAP1* GPV carriers via large gene panels. It is important to consider the European model of healthcare, in which services are, in general, funded through taxes and/or social contributions. Thus, guidelines need to consider both the cost and burden on healthcare systems, as well as the balance of desirable versus undesirable outcomes and quality of evidence.

To address these issues, the Clinical Guideline Working Group of the CanGene-CanVar project in the United Kingdom invited European collaborators to join them to develop guidelines for *BAP1* carriers to harmonize surveillance programmes within Europe. The recommendations are summarised in this report.

### Scope of the guideline

The overarching aim of the guidance is to provide a resource to healthcare professionals to aid in the identification and surveillance of individuals with *BAP1*-TPDS by considering a number of key questions (Table 1). It was decided that the management and treatment of specific tumours associated with *BAP1* GPV and assessment of *BAP1* variants of uncertain significance was outside the scope of this guideline. It is important to note that these guidelines do not represent a legal standard of care, and whilst they should support clinical practice, they should not replace clinical judgement.

**Table 1:** Key questions.

Questions
***BAP1*** **phenotypic spectrum**
What cancers can be considered part of the *BAP1*-tumour predisposition syndrome?
Are there specific histological subtypes of associated cancers?
Are there other cancers whose association is not yet established?
***BAP1*** **cancer risk**
What are the lifetime cancer risks for *BAP1* carriers, overall and for specific cancers?
**Identification of** ***BAP1*** **carriers**
What is the detection rate of *BAP1* GPV in patients with a specific cancer?
Who should be offered genetic testing of *BAP1*?
***BAP1*** **cancer surveillance**
Should individuals undergo surveillance for mesothelioma, renal cancer, uveal or cutaneous melanoma or other cancers? If so, what are the recommendations- clinical or research?
***BAP1*** **predictive testing (i.e., testing an unaffected individual for a familial GPV)**
What age should predictive testing be recommended?
What surveillance should be offered to those at 50% risk?

## Methods

CanGene-CanVar (a Cancer Research UK funded research group) commissioned the guidelines and invited clinicians from the UK and Europe to participate in the expert clinical group. The core group consisted of 10 members and an extended expert group of 34 additional members. Members of the group were invited due to a known interest in this area and via email invitation to the European Reference Network for genetic tumour risk syndromes (ERN GENTURIS) group to ensure that relevant clinical specialties were represented, including Genetics, Ophthalmology, Oncology, Dermatology and Pathology.

The scope and purpose of the guidelines were initially discussed in November 2020, following which a set of key questions were formulated (Table 1).

The core group members had six virtual meetings between November 2020 and June 2022 to discuss the questions, agree on methodology and allocate specific topics to each member of the core group. A literature search was undertaken in January 2021 with predefined search terms, using PUBMED and subsequent extended searches via citation chasing and limiting to articles in English. Evidence levels and recommendations were assessed using a modified GRADE system as utilised by the ERN GENTURIS network in *PTEN* Hamartoma Tumour Syndrome (PHTS) guideline development [7]. The questions were reviewed and initial draft statements formulated by the core group to be reviewed by the extended group via a modified electronic Delphi process methodology [8]. Delphi is a structured process in which opinions of a large number of experts are gathered on statements for which there is limited or weak evidence. Each statement was scored by each member of the group using a five-point scale (strongly disagree to strongly agree). To achieve consensus, at least 80% agreement of respondents to that statement was required. Where there was disagreement and an 80% consensus not reached, the comments and discussion from the Delphi group were used to amend recommendations for a subsequent round of Delphi. If consensus was not reached after two rounds, the statement was excluded from the final guidelines. Even if consensus was met, the statements were still modified if comments indicated clearer wording or format. The results of the Delphi survey were reviewed by the core group to agree the final statements and recommendations.

## Results

As is common within the rare disease field, there is limited peer-reviewed evidence available to inform guideline development with only limited numbers of *BAP1* carriers reported in the literature (Supplementary Table 2) and scant evidence addressing surveillance within this patient group.

In round 1 of the Delphi, 29 statements reached consensus (80% or greater consensus) and 13 did not. For Delphi round 2, 23 statements were put forward (13 that did not achieve consensus and 10 reworded following comments on clarity or consistency). At the end of round 2, consensus was reached on all but four statements. The final recommendations are detailed in Table 2a–d, with the evidence weighting using the modified GRADE system outlined in the ERN GENTURIS PHTS guidelines [7] (Strong evidence: Consistent evidence and new evidence unlikely to change recommendation and expert consensus; Moderate evidence: Expert consensus or majority decision but with inconsistent evidence or significant new evidence expected and weak evidence: inconsistent evidence and limited expert agreement).

**Table 2:** (a) Phenotype of *BAP1*-tumour predisposition syndrome. (b) Prevalence of *BAP1* germline pathogenic variants. (c) Recommendations for germline *BAP1* testing. (d) Recommendations for surveillance of *BAP1* carriers.

	Consensus	Strength of recommendation
**(a)**		
**Phenotype of** ***BAP1*-tumour predisposition syndrome** (***BAP1*-TPDS**)		
Mesothelioma is part of the core phenotype (defined as occurring in 10% or more of carriers) in *BAP1*-TPDS	*n* = 42; 31 strongly agree, 11 agree, 100% consensus	Strong
Renal cell carcinoma is part of the core phenotype (defined as occurring in 10% or more of carriers) in *BAP1*-TPDS	*n* = 42; 19 strongly agree, 15 agree, 81% consensus	Moderate
Uveal melanoma is part of the core phenotype (defined as occurring in 10% or more of carriers) in *BAP1*-TPDS	*n* = 42; 34 strongly agree, 8 agree, 100% consensus	Strong
Cutaneous melanoma is part of the core phenotype (defining as occurring in 10% or more of carriers) in *BAP1*-TPDS	*n* = 42; 24 strongly agree, 17 agree, 98% consensus	Strong
Cutaneous melanocytic lesions, BAP1 inactivated melanocytoma - BIM (BAP-oma, atypical Spitz tumour, melanocytic BAP1 associated intradermal tumour, BAP1 inactivated melanocytic nevus) are part of the core phenotype (occur in 10% or more of carriers) in *BAP1*-TPDS	*n* = 42; 26 strongly agree, 12 agree, 90% consensus	Strong
Meningioma can be considered part of the *BAP1* associated tumour spectrum but should not be considered as part of the core phenotype	*n* = 42; 7 strongly agree, 29 agree, 86% consensus	Moderate
Hepatocellular carcinoma has been suggested to be part of the *BAP1* associated tumour spectrum, but further evidence is required to confirm an association	*n* = 37; 5 strongly agree, 28 agree, 89% consensus	Moderate
Cholangiocarcinoma has been suggested to be part of the *BAP1* associated tumour spectrum, but further evidence is required to confirm an association	*n* = 37; 34 strongly agree, 3 agree, 91% consensus	Moderate
Other cancers including breast and lung cancer should not currently be considered to be part of the *BAP1* tumour predisposition syndrome	*n* = 42, 12 strongly agree, 25 agree, 88% consensus	Moderate
**(b)**		
**Prevalence of** ***BAP1*** **pathogenic variants**		
The prevalence of germline *BAP1* pathogenic variants in individuals with isolated mesothelioma is low (less than 3%)	*n* = 37; 7 strongly agree, 23 agree, 81% consensus	Strong
The prevalence of germline *BAP1* pathogenic variants in individuals with isolated renal cell carcinoma is low (less than 3%)	*n* = 37; 7 strongly agree, 27 agree, 92% consensus	Strong
The prevalence of germline *BAP1* pathogenic variants in individuals with isolated uveal melanoma is low (less than 3%)	*n* = 37; 6 strongly agree, 26 agree, 86% consensus	Strong
The prevalence of germline *BAP1* pathogenic variants in individuals with isolated cutaneous melanoma is low (less than 3%)	*n* = 37; 13 strongly agree, 22 agree, 95% consensus	Strong
Individuals with two or more *BAP1*-associated core tumours or a typical BAP1-associated core tumour and a family history have a moderate to high prevalence of *BAP1* germline pathogenic variants (>10% detection rate)	*n* = 42; 13 strongly agree, 25 agree, 91% consensus	Moderate
**(c)**		
**Individual indications**		
Germline *BAP1* genetic testing should be offered to all individuals with a personal history of *two or more* core *BAP1* associated tumours (mesothelioma, uveal melanoma, cutaneous melanoma, renal cell cancer or *BAP1* inactivated melanocytic tumour- BIM) (excluding two cases of melanoma)	*n* = 37; 18 strongly agree, 16 agree, 91% consensus	Strong
Germline *BAP1* genetic testing should be offered to all individuals with a personal history of *two or more* inactivated melanocytic tumours (BIM)	*n* = 42; 19 strongly agree, 19 agree, 91% consensus	Strong
Germline *BAP1* genetic testing should not routinely be offered to all individuals diagnosed with isolated cases of uveal melanoma, mesothelioma, cutaneous melanoma or renal cell carcinoma, but can be considered based on age of onset and other factors such as absence of asbestos exposure or tumour results (see further cancer specific recommendations below)	*n* = 37; 10 strongly agree, 25 agree, 95% consensus	Moderate /weak
Germline *BAP1* genetic testing *may* be considered in isolated young onset mesothelioma (less than 60 years) in the absence of asbestos exposure	*n* = 37; 9 strongly agree, 21 agree, 81% consensus	Moderate
Germline *BAP1* genetic testing *may* be considered in isolated young onset cases of renal cancer as part of a larger germline cancer predisposition gene panel	*n* = 37; 7 strongly agree, 28 agree, 95% consensus	Moderate
Germline *BAP1* genetic testing *may* be considered in isolated cases of uveal melanoma (<40 years)	*n* = 37; 9 strongly agree, 25 agree, 92% consensus	Moderate
Germline *BAP1* genetic testing *may* be considered for isolated childhood cases of cutaneous melanoma (less than 18 years) as part of a larger germline cancer predisposition gene panel	*n* = 37; 10 strongly agree, 25 agree, 95% consensus	Moderate
Individual plus family indications		
Germline *BAP1* genetic testing should be offered to all individuals with a personal history of a *BAP1* associated tumour *and* a first degree relative with a *BAP1* core associated tumour (mesothelioma, uveal melanoma, cutaneous melanoma, renal cell cancer or *BAP1* inactivated melanocytic tumour- BIM) (excluding two cases of melanoma)	*n* = 37; 15 strongly agree, 18 agree, 91% consensus note consensus was not achieved for situation of proband with only a SDR affected with core tumour	Moderate
Tumour to germline testing		
Where a *BAP1* pathogenic variant (as defined by ACMG/AMP criteria) has been identified in a *BAP1*-associated tumour, germline testing may be considered based on variant allele frequency, tumour type and other personal or family history suggestive of *BAP1*-TPDS following discussion in a molecular or genomic tumour board	*n* = 37; 7 strongly agree, 28 agree, 95% consensus	Moderate
Germline *BAP1* genetic testing should not routinely be offered to individuals where a *BAP1* pathogenic variant (as defined by ACMG/AMP criteria) has been identified in a tumour not associated with *BAP1* associated tumour syndrome (Off-tumour setting), unless there is an additional personal of family history of *BAP1* associated cancers	*n* = 42; 8 strongly agree, 26 agree, 90% consensus	Moderate
Testing for familial variant		
Predictive genetic testing for a familial *BAP1* variant should only be offered where the variant has been classified as “likely pathogenic” or “pathogenic” according to ACMG/AMP criteria	*n* = 42; 21 strongly agree, 13 agree, 81% consensus	Strong
Predictive genetic testing for a known familial pathogenic variant in *BAP1* should be offered from the age of 16–18 years	*n* = 42; 7 strongly agree, 30 agree, 88% consensus	Moderate
**(d)**		
**Surveillance for** ***BAP1*** **carriers**		
Annual renal imaging should be offered to all *BAP1* carriers	*n* = 40, 10 strongly agree, 24 agree, 85% consensus	Weak
The preferred modality of renal imaging is MRI. If unavailable, renal ultrasound scan can be used as an alternative. MRI is recommended for ongoing surveillance if a lesion requiring follow up is detected	*n* = 39, 6 strongly agree, 26 agree, 82% consensus	Weak
Annual renal imaging should start from age 30 years	*n* = 40, 4 strongly agree, 28 agree, 80% consensus	Weak
At present, there are no data to suggest that surveillance for mesothelioma (with either chest X-ray or MRI) has the ability to detect mesothelioma at an early stage. Prospective studies of surveillance in *BAP1* carriers may help answer this question and, therefore, at present surveillance for mesothelioma should be undertaken in the setting of a research study	*n* = 37, 6 strongly agree, 24 agree, 81% consensus	Moderate
Annual dilated ophthalmic examination should be offered to all *BAP1* carriers by an ocular oncologist or an ophthalmologist with an expertise in uveal melanoma	*n* = 40, 19 strongly agree, 20 agree, 98% consensus	Moderate
Annual ocular examination should start from 16–18 years	*n* = 40, 9 strongly agree, 26 agree 88% consensus	Weak
Annual dermatological review including full body examination and photography should be offered to all *BAP1* carriers	*n* = 41, 12 strongly agree, 22 agree, 83% consensus	Moderate
Annual dermatology review should start from age 18–20	*n* = 41, 8 strongly agree, 27 agree, 83% consensus note consensus not reached for self-examination	Weak
For clinically suspected BIM, if the numbers are small, excision should be reserved for clinically suspicious lesions (growing/changing) or where there is diagnostic uncertainty	*n* = 36, 3 strongly agree, 21 agree, 88% consensus	Weak

### Rationale for the recommendations

#### Phenotype and prevalence of germline *BAP1* pathogenic variants

In 2016 Rai et al. undertook a literature review to assess the phenotypic spectrum of *BAP1* –TPDS [5]. They noted that 31% (54/174) of *BAP1* carriers were diagnosed with a uveal melanoma, 22% (39/174) had a malignant mesothelioma (26/39 of which were pleural), 13% (23/174) of cases had a cutaneous melanoma and 10% (18/174) had renal cell carcinoma. A subsequent comprehensive literature review of 181 families with GPV in *BAP1* by Walpole et al. [2] assessed cancers associated with *BAP1* GPV and suggested that mesothelioma (peritoneal and pleural), uveal melanoma, cutaneous melanoma, and renal carcinoma are core cancers in the phenotype, occurring in 24.5%, 36.2%, 23.4%, and 5.7% of 141 proband carriers and 16.9%, 15.9%, 12.0%, and 4.9% of 183 non-proband carriers respectively. *BAP1*-inactivated melanocytic tumours (BIM) have since been considered to be a feature of *BAP1*-associated tumour syndrome, identified in up to 72% of cases [9, 10]. Other cancers proposed to be associated with *BAP1* GPV include meningioma, hepatocellular carcinoma, cutaneous basal cell cancer and cholangiocarcinoma [11–13].

In agreement with Walpole et al. [2], consensus was achieved that mesothelioma (peritoneal and pleural), uveal melanoma, cutaneous melanoma, and renal carcinoma, along with BIM are core cancers associated with *BAP1*-TPDS. The group also agreed that the evidence supports an association for meningioma, but that it should not be considered part of the core phenotype. Further evidence was required to confirm an association for hepatocellular carcinoma, cholangiocarcinoma or other cancers (Table 2a).

The group also discussed the potential association of specific histological subtypes of cancer with *BAP1* GPV, specifically whether rhabdoid compared to non-rhabdoid meningioma is more strongly associated as suggested by some studies [14] or whether a specific type of renal cancer is associated with *BAP1* GPV. After two Delphi rounds, consensus was not achieved, and it was felt that further studies are required to support an association of specific histological subtypes.

#### Prevalence of *BAP1* pathogenic variants

Population studies of patients with cutaneous melanoma, ocular melanoma, malignant mesothelioma, and renal cell carcinoma identified *BAP1* GPV in less than 1% of patients [15–17]. Subsequent studies have refined the criteria to improve detection rates, using additional information from tumour based testing or personal/family history of *BAP1*- associated tumours.

The Danish registry study [18] demonstrated a 25% detection rate in cutaneous melanoma families with two or more cases of uveal melanoma as well as cutaneous melanoma. Familial uveal melanoma is far more likely to be due to *BAP1* GPV than sporadic cases with a pick up rate of 25% [19].

Panou et al. [20] undertook mutation testing in 195 cases of mesothelioma but only identified six *BAP1* GPV. Of these, four cases had a family history of a *BAP1*-associated tumour, one had personal history of another malignancy and one was an isolated case, again demonstrating a higher detection rate in the presence of relevant personal or family history.

The probability of identifying a GPV in *BAP1* is also low in most cases of renal cell carcinoma (RCC). Popova et al. [21] described the association of RCC with *BAP1* GPV in 2013 in a family with multiple cancer types and four first-degree relatives with RCC; however, on evaluation of 32 other RCC families they failed to identify any further *BAP1* GPV. Other studies assessing familial cases of RCC also failed to identify any GPV [22]. A large study of 254 cases with advanced renal cell carcinoma identified *BAP1* GPV in 1% (3/254) of cases. All of these tumours demonstrated loss of *BAP1* on IHC [23]. There is some suggestion that early onset cases may have an increased pick up rate although Wu et al. [24] only identified 3 *BAP1* GPV in 190 cases (1.6%) diagnosed under 45 years of age.

Cabaret et al. [9] identified *BAP1* GPV in 24% (12/49) of individuals with BIM demonstrating loss of *BAP1* on immunohistochemistry (IHC). The majority of the patients with a BIM demonstrating loss of *BAP1* on IHC and a *BAP1* GPV had a personal or family history of any other *BAP1* associated tumour (10/12 cases).

Meningioma has been proposed as part of *BAP1*-TPDS. However, the data around the prevalence of mutations are limited. Shankar et al. [11] demonstrated a detection rate of *BAP1* GPV of 3% in the rhabdoid subtypes (an uncommon aggressive form). However, rhabdoid subtypes are rare and thus the evidence is limited and consensus was not achieved for an association with a particular subtype.

Likewise, cholangiocarcinoma has been investigated due to the high somatic mutation rate in *BAP1* in these tumours [12, 25]. However, only a single *BAP1* GPV was identified in 131 cases (0.7%) in an individual with a family history of *BAP1*- associated tumours. Other germline cases described have been in individuals with a personal or family history of *BAP1*-associated tumours [13, 26].

Conversely, somatic variants in *BAP1* are common in a number of malignancies. Carbone et al. [27] reviewed the literature and stated that somatic variants of *BAP1* were identified in 60–70% of mesotheliomas, 45% of uveal melanomas, 15% of renal cell carcinomas, and 5% of cutaneous melanomas. Somatic *BAP1* mutations have also been identified in other malignancies, including cholangiocarcinoma and thymic cancers. A recent paper has confirmed these somatic variant rates [28].

#### *BAP1* germline testing indications

As for many other cancer predisposition genes, existing *BAP1* testing criteria have generally been based on personal history of *BAP1*-TPDS associated tumours or a personal and family history of *BAP1*-TPDS associated tumours [5]. More recent guidelines have suggested the addition of young age of onset of a *BAP1*-associated tumour [4]. Whilst in general, thresholds for genetic testing are decreasing due to diminishing costs and labour of genetic testing, most European countries apply a threshold at which genetic testing can be offered. In the UK and other European countries this has historically been set at approximately 10%, with the exception of genes where there are therapeutic implications for cancer management and/or clear risk reducing interventions, where lower thresholds may be considered.

The implementation of next generation sequencing gene panels, facilitating testing for specific indications have resulted in much broader eligibility criteria e.g., panels for inherited renal cancer or familial melanoma https://panelapp.genomicsengland.co.uk/panels/522/https://panelapp.genomicsengland.co.uk/panels/521/. Increasing implementation of large solid tumour panel tests and paired whole genome sequencing means that *BAP1* testing may also be undertaken in patients with cancer outside the known spectrum of *BAP1*-TPDS. At present, disease causing *BAP1* variants are not on the list of ACMG actionable secondary findings due to concerns around penetrance and management of carriers in the context of identification in unrelated conditions [29].

As described in the prevalence section, the detection rate for a germline *BAP1* pathogenic variant is higher where there is a personal or family history of other *BAP1*-associated tumours and lower for isolated cases. The group felt that targeted germline testing of *BAP1* was most relevant in the situation of a strong personal or family history of *BAP1*-associated tumours, whereas in the case of an isolated *BAP1*-related tumour without other suggestive features, where the detection rate is lower, testing may be indicated based on country specific guidelines or may be more appropriate in the setting of a larger panel.

Due to the high somatic rate of *BAP1* variants, it was felt that reflex *BAP1* germline testing was not appropriate and the decision to proceed to germline testing should only be undertaken in the context of a *BAP1*-associated tumour with additional supportive information e.g., IHC loss, age of onset and personal /family history. This is consistent with the paper by Kuzbari et al. [30], suggesting that if a threshold of 10% for detection of germline variants following somatic variant detection is recommended, *BAP1* germline testing should not be undertaken.

Predictive genetic testing (i.e., genetic testing for a known familial variant) should only be offered if the familial variant in *BAP1* is likely pathogenic or pathogenic according to ACMG/ACP guidelines [31]. Offering predictive testing was felt to be appropriate in late teens when surveillance may start (see next section). There may be situations where testing family members for a variant of unknown significance (VUS) is helpful for the purpose of segregation studies if this helps in the evaluation of the pathogenicity of the variant. Also, when awaiting the outcome of functional testing in a research setting for a VUS, it may be appropriate to consider dermatologic and ophthalmic screening in first degree relatives, but this may be best decided in the context of specialist MDT discussion.

#### Cancer risks and surveillance for *BAP1*-associated cancer

The malignancy risk associated with germline *BAP1* pathogenic variants is unclear due to limited data on long term follow up of individuals ascertained via cascade testing following a diagnostic test in an affected individual. Due to ascertainment bias, the current estimates of risk are likely to be overestimated. A summary of published studies are detailed in Supplementary Table 2.

The largest reported study of *BAP1*-TPDS carriers (*n* = 181 families) suggests that lifetime cancer risks were reported to be different for truncating compared to missense variants [2]. The frequency of cancers reported in probands with truncating and missense variants for uveal melanoma was 36% (36/141) and 23% (9/40), for melanoma 23% (33/141) and 45% (18/40), for malignant mesothelioma 25% (35/141) and 15% (6/40), and for renal cell cancer 6% (8/141) and 10% (4/40). The frequency of cancers described for the non-probands were lower suggesting ascertainment bias. The age of onset of the cancers is younger than in the general population with an estimated worse survival of cases of uveal melanoma, cutaneous melanoma and renal cell carcinoma. Conversely, there is some evidence that individuals with mesothelioma and *BAP1 GPV* have a better survival [32].

### Uveal melanoma

Uveal melanoma (UM) is the most frequently occurring primary malignancy in the eye of adults. UM is generally assumed not to be a hereditary disease, though the occurrence of bilateral UM is more common than predicted by chance alone [33]. Furthermore, about 1–6% of patients with UM has a family history of this disease [16, 34]. A fraction of these cases can be explained by *BAP1*-TPDS [19].

In the largest described cohort to date, the occurrence of UM in probands of *BAP1*-TPDS families is 51/141 (36.2%). The occurrence in non-proband variant carriers is 29/183 (15.9%) [2]. A more recent study has estimated the point prevalence of 2.8% (95% CI 0.88–4.81%) of UM in patients with *BAP1*-TPDS, compared to 0.0061% in Non-Finnish population (95% CI 0.0058–0.0063%) [35]. Whilst it is clear that patients with *BAP1*-TPDS have an increased risk of developing UM relative to the general population, the penetrance of UM in *BAP1*-TPDS has not yet been completely elucidated.

Patients with *BAP1*-TPDS develop UM at a younger age compared to the patients with incidental UM [2]. Of the approximately 100 published cases of UM in *BAP1*-TPDS, the youngest patient was 15 years old. However, imaging suggested ocular melanocytosis in this patient, which is another risk factor for developing UM [25, 36]. Patients with oculo(dermal) melanocytosis have an estimated life time risk of 1/400 (0.0025%) of developing UM [8, 37]. Other studies suggest that the median age of onset of uveal melanoma in *BAP1*-TPDS is in the 6th decade [2, 38].

There was strong consensus from the group that regular surveillance for UM is indicated. Ophthalmological examination is non-invasive and relatively simple to perform. There is also evidence that smaller local lesions have a significantly improved prognosis compared to metastatic disease and that treating high risk smaller lesions will improve mortality [39, 40]. Surveillance may therefore lead to improved visual outcomes and better survival if UM is diagnosed at an earlier stage. Future prospective studies are needed to prove this assumption. There are few data about the required frequency of surveillance.

### Skin tumours

The background incidence of malignant melanoma depends on a variety of genetic and environmental factors including skin type and sun exposure. There are a number of genetic conditions that can predispose to cutaneous melanoma with clearly established screening strategies. It has been long demonstrated that complete cutaneous examination by dermatologists increases the diagnosis of early malignant melanoma [41].

Whilst there is a clear association of cutaneous melanoma and BIM with *BAP1* GPV, the exact risk of cutaneous malignancy is unclear.

Star et al. [6] postulated guidelines for *BAP1* tumour surveillance which suggested that high risk melanoma surveillance be tailored for individuals with *BAP1* GPV. There are data to suggest that self-examination increases the rate of diagnosis of early lesions in families with increased risk of melanoma that may be relevant to *BAP1* GPV carriers [42, 43]. The group reached consensus that annual dermatological review including full body examination and photography should be offered to all *BAP1* carriers, but consensus was not reached about the frequency of self-examination.

According to expert opinion, the transformation risk of *BAP1*-inactivated melanocytic nevi/tumours into melanoma is low, similar to atypical/dysplastic nevi (Clark nevi). Thus, the suggested clinical approach would be similar to Clark nevi. *BAP1*-inactivated melanocytic nevi/tumours that are clinically suspicious for melanoma following assessment by a dermatologist should be excised, Pre-emptive excision of all *BAP1*-inactivated melanocytic nevi/tumours may not be feasible, and the benefit of this approach remains unclear.

### Mesothelioma

Mesothelioma is a rare tumour with European standardised incidence rates of 1.7 per 100,000 men and 0.4 per 100,000 women. There is a clear association with asbestos exposure but, even in those areas with very high exposure, the lifetime estimate is only about 5%. There is also a clear association with age, with risks increasing substantially from the age of 50 years. The median survival from mesothelioma is 9–12 months [44].

The lifetime risk of mesothelioma for *BAP1 GPV* carriers has been estimated at between 15–25% [2]. Mesothelioma is recognised as an aggressive tumour with a poor prognosis, most typically caused by asbestos exposure. To date, no surveillance programme for mesothelioma has been implemented, even for those known to be at high risk due to environmental exposure. However, there is some evidence to suggest that mesothelioma occurring as a result of a *BAP1 GPV*, may be associated with better survival compared to population-based cases [32, 45, 46]. Given the improved survival, the guideline group discussed that surveillance may be of use as this may allow earlier intervention. However, there are no published studies and the modality (e.g., (computed tomography [CT], magnetic resonance imaging [MRI]) and frequency of surveillance is unclear. Therefore, it was considered that surveillance for mesothelioma may only be appropriate in a research setting that allows prospective data collection and study of the long-term outcomes of screening. The U.S. National Cancer Institute (NCI) has opened two clinical trials to prospectively study frequency of mesotheliomas and other cancers in individuals with *BAP1* GPV, which may address some of these questions [46].

### Renal tumours

Randomised control trials of renal surveillance in *BAP1* carriers have not been undertaken. Most studies have identified the frequency of renal cancer in germline *BAP1* carriers, and in many studies the type of renal cancers have not been histopathologically verified. There have been reports of both multifocal and bilateral RCC in *BAP1* carriers, and the average age of onset is around 10 years younger than in sporadic RCC.

However, multiple studies have shown that somatic loss of *BAP1* in renal cell carcinoma is associated with a more aggressive clinical behaviour and worse prognosis [47].

In addition, a single small study of six tumours from two patients demonstrated an increased growth rate of tumours in *BAP1* carriers [48]. The age at which renal cancer is diagnosed is lower in individuals with a pathogenic variant in *BAP1* with a range of between 32–80 years.

Ultrasound scans are low-cost and easily available. However, whilst 77% of RCC < 30 mm are in general hyperechoic and detectable by ultrasound, a proportion are not detected [49]. The detection of isoechoic or hypoechoic tumours is more challenging, and whilst there are currently no data available regarding whether *BAP1* tumours are hyperechoic, MRI could be considered as the preferred surveillance modality. This would be consistent with the recommendation for patients with hereditary leiomyomatosis and renal cell carcinoma (HLRCC) who are at risk of more aggressive renal tumours.

Consensus was reached that annual renal imaging should be offered to all *BAP1* carriers, and that the preferred modality of imaging would be MRI. However, USS could be used as an alternative should MRI not be available. Given the reported age of onset of renal cancer in *BAP1* carriers, starting from age 30 years reached consensus. It was recognised, as for the other tumours that long term data collection is required to evaluate the long-term benefit of surveillance.

#### Other recommendations for *BAP1* carriers

Whilst not specifically discussed as part of the Delphi process, the core group considered that *BAP1* carriers should be given appropriate lifestyle advice, including but not limited to avoiding occupations with asbestos exposure, being aware of residential exposure during renovations, non-smoking and limiting UV exposure.

## Discussion

As with many rare conditions, data around management are limited. *BAP1* GPV are now being identified not only in individuals with *BAP1*-associated tumours, but also as incidental findings as part of larger gene panels.

The currently established risk figures for malignancies associated with GPV in *BAP1* are likely to be overestimates due to ascertainment bias in the reported series and long term follow up over many years will be required in order to more accurately determine risks. By virtue of the small numbers of individuals affected, randomised trials of surveillance will never be possible and long-term, prospective observation of patients undergoing surveillance is to be recommended.

These guidelines are a pragmatic approach, based on the best current available evidence along with expert opinion. We recognise the limitations of this approach, given the limited experience each individual clinician has with families with *BAP1* GPV. However, a Delphi process allows for discussion with a wide group of experts in a number of clinical fields. This approach also enables consideration of the healthcare systems across Europe.

### Supplementary information

Supplementary table 1

Supplementary table 2

## Data Availability

All data generated or analysed during this study are included in this published article [and its supplementary information files].
