# Supplementary material for: Clinical practice guidelines for the diagnosis and surveillance of BAP1 tumour predisposition syndrome
Source: Eur J Hum Genet. 2023 Aug 22;31(11):1261–9. doi: 10.1038/s41431-023-01448-z (PMC10620132; doi:10.1038/s41431-023-01448-z)
Supplement: Supplementary file 1 — Supplementary table 1 [file 41431_2023_1448_MOESM1_ESM.docx]

**Supplementary Table 1: Summary of current recommendations for *BAP1* carriers**

|  | **American recommendations ^1^** | **Australian recommendations ^2^** | **Dutch National recommendations (personal communication M.Nielsen)** | **Gene Reviews (Last update March** **https://www.ncbi.nlm.nih.gov/books/NBK390611/ 2022)** |
| --- | --- | --- | --- | --- |
| **Lung** | Annual physical examination | Annual physical examination from age 30 | Yearly MRI thorax (research)  Review by Oncology Pulmonologist | Annual physical examination.  If using MRI of abdomen, evaluate peritoneum and pleura as well |
| **Kidney** | Annual USS and two-yearly MRI from age 30 | Two-yearly USS or MRI from age 30-55, then CT/MRI from age 55 | Yearly MRI abdomen (research)  Yearly annual USS | Two yearly USS and 2 yearly MRI alternating from 30 years (i.e annual examination) |
| **Skin** | Yearly exam from age 20 (Dermatology – full body) | 6 monthly exams from age 18 (Dermatology – full body exam and photography) | Yearly exam from age 16 (Dermatology)  Monthly self check | Annual examination from 18 years by dermatology, consider whole body imaging if there are a large number of lesions |
| **Eyes** | Yearly exam from age 11 (Ophthalmology -dilated eye exam and imaging) | Yearly exam from age 16 annually and from 30 6 monthly (Ophthalmology – dilated eye exam, fundus photography, ocular USS) | Yearly exam from age 16 (Ophthalmology) | Annual dilated eye examination by ocular oncologist or general ophthalmologist with appropriate referral pathways |

1. Rai K, Pilarski R, Cebulla CM, Abdel-Rahman MH. Comprehensive review of BAP1 tumor predisposition syndrome with report of two new cases. Clin Genet. 2016;89(3):285–94.

2. Star P, Goodwin A, Kapoor R, Conway RM, Long G V., Scolyer RA, et al. Germline BAP1-positive patients: the dilemmas of cancer surveillance and a proposed interdisciplinary consensus monitoring strategy. Eur J Cancer [Internet]. 2018;92:48–53. Available from: https://doi.org/10.1016/j.ejca.2017.12.022
