# Supplementary material for: Clinical practice guidelines for the diagnosis and surveillance of BAP1 tumour predisposition syndrome
Source: Eur J Hum Genet. 2023 Aug 22;31(11):1261–9. doi: 10.1038/s41431-023-01448-z (PMC10620132; doi:10.1038/s41431-023-01448-z)
Supplement: Supplementary file 2 — Supplementary table 2 [file 41431_2023_1448_MOESM2_ESM.docx]

**Supplementary Table 2 :Tumour types identified in *BAP1* carriers**

| **Tumour Type** | **Lifetime Risk** | **Lifetime risk (non probands)** | **Range of ages in the literature** | **Total cases in literature** | |
| --- | --- | --- | --- | --- | --- |
|  |  |  |  | **Reference** | **numbers** |
| **Uveal Melanoma** | 25% | Singh et al 2020 point prevalence risk 2.8%  Chau^6^ estimated disease free proportion at 65 years in non-probands of 0.93 (95% CI 0.79–1)  Walpole^1^ gave tumour frequencies in cohort –overall 11% | 15-79yrs | Walpole 2018^1^ | 92 |
|  |  |  |  | Yu 2020 ^2^ | 2 |
|  |  |  |  | Young 2020^3^ | 1 |
|  |  |  |  | Yaghy 2019^4^ | 1 |
|  |  |  |  | Chau 2019^5^ | 9 |
|  |  |  |  | Repo 2019^6^ | 3 |
|  |  |  |  | Pastorino 2018^7^ | 7 |
|  |  |  |  |  |  |
| **Mesothelioma** | 20% | Chau^6^ estimated disease free proportion at 65 years in non-probands of 0.74 (95% CI 0.54–0.95)  Walpole^1^ gave tumour frequencies in cohort –overall 13.3%* | 39-71yrs*** | Walpole 2018^1^ | 68 |
|  |  |  |  | Yoshikawa 2020^8^ | 3 |
|  |  |  |  | Malpica 2020 | 1 (peritoneal) |
|  |  |  |  | Yaghy 2019^4^ | 1 |
|  |  |  |  | Shinozaki-Ushiku 2019^9^ | 1 |
|  |  |  |  | Chau 2019^5^ | 8 (2 peritoneal) |
|  |  |  |  | Zauderer 2019^10^ | 5 (1 peritoneal) |
|  |  |  |  | Pastorino 2018^7^ | 43 |
|  |  |  |  |  |  |
| **Cutaneous melanoma** | 20% | Chau^6^ estimated disease free proportion at 65 years in non-probands of 0.85 (95% CI 0.73–0.97)  Walpole^1^ gave tumour frequencies in cohort –overall 10% | 23-83yrs*** | Walpole 2018^1^ | 63 |
|  |  |  |  | Costa soares de Sa 2019^11^ | 1 |
|  |  |  |  | Chau 2019^12^ | 13 |
|  |  |  |  | O’Shea 2017^13^ | 3 (loss of function mutations only) |
|  |  |  |  | Pastorino 2018^7^ | 3 |
|  |  |  |  |  |  |
| **Renal cancer (clear cell, papillary and chromophobe)** | 5-10% | Chau^6^ estimated disease free proportion at 65 years in non-probands of 0.94 (95% CI 0.84–1)  Walpole^1^ gave tumour frequencies in cohort –overall 5.3% | 36-69yrs | Walpole 2018^1^ | 22 |
|  |  |  |  | Walpole 2018^1^ | 2 |
|  |  |  |  | Pastorino 2018^7^ | 5 |
|  |  |  |  | Costa soares de Sa 2019^11^ | 1 |
|  |  |  |  | Chau 2019^12^ | 13 |
|  |  |  |  | O’Shea 2017^13^ | 3 (loss of function mutations only) |
|  |  |  |  | Pastorino 2018^7^ | 3 |
|  |  |  |  |  |  |
| **Cutaneous melanocytic lesions (BAP-oma, atypical Spitz tumor, melano- cytic BAP1-associated intradermal tumor, nevoid melanoma- like proliferation, or BAP1-inactivated melanocytic nevus/mela- nocytoma)** | - | **-** | Median 14-55yrs | Young 2020^3^ | 1 |
|  |  |  |  | Amber 2020 | 1 |
|  |  |  |  | Chau 2019^12^ | 8 |
|  |  |  |  | Satolli 2019^14^ | 1 |
|  |  |  |  | Costa soares de Sa 2019^11^ | 1 |
|  |  |  |  | Wiesner 2011^15^ | 5 |
|  |  |  |  | Louie 2020^16^ | 40 |
|  |  |  |  | Young 2020^3^ | 1 |
|  |  |  |  | Amber 2020 | 1 |
|  |  |  |  |  |  |
| **Cholangiocarcinoma** | 1% | Walpole^1^ gave tumour frequencies in cohort –overall 0.6% | 47-72yrs | Walpole 2018^1^ | 4 |
|  |  |  |  | Brandi 2020^17^ | 1 |
|  |  |  |  | Maynard 2020^18^ | 1 |
|  |  |  |  | Shinozaki-Ushiku 2019^9^ | 1 |
|  |  |  |  |  |  |
| **Non-melanoma skin tumours (BCC and SCC)** | 7% | Walpole^1^ gave tumour frequencies in cohort –overall 7.2 | 27-66 | Walpole 2018^1^ | 27 |
|  |  |  |  | Chau 2019^12^ | 19 |
|  |  |  |  | Pastorino 2018^7^ | 5 |
|  |  |  |  |  |  |
| **Hepatocellular carcinoma** | NK | NK | 49-68 | Chau 2019^12^ | 3 |
|  |  |  |  | Pastorino 2018^7^ | 1 (? Pathology) |
|  |  |  |  |  |  |
| **Lung*** | NK | NK | 43-49 | Walpole 2018^1^ | 13 |
|  |  |  |  | Shinozaki-Ushiku 2019^9^ | 1 |
|  |  |  |  | Pastorino 2018^7^ | 1 |
|  |  |  |  |  |  |
| Breast** | NK | NK | 45-58 | Walpole 2018^1^ | 21 |
|  |  |  |  | Pastorino 2018^7^ | 6 |
|  |  |  |  |  |  |
| Others (Colorectal, ovarian, sarcoma, prostate, bladder, stomach) | NK | NK | NK | Walpole 2018^1^ | 19 |
|  |  |  |  | Abdel Rahman 2011^19^ | 1 |
|  |  |  |  | Dumbrava 2019^20^ | 1 |
|  |  |  |  | Pastorino 2018^7^ | 3 |
|  |  |  |  | Tesch 2020^21^ | 2 |
|  |  |  |  | Shinozaki-Ushiku 2019^9^ | 1 |
|  |  |  |  |  |  |

*unclear from the studies if definite new primary and metastatic disease excluded

** rate of breast cancer described the same as general population rate

Walpole likely to be an overestimate as included those untested individuals within a family

and in order to be included needed to have at least one tumour

*** Carbone et al. report younger ages of onset but from personal communication rather than published papers^22^ (cutaneous melanoma -age 8 and peritoneal malignant mesothelioma age 28)

(1) Walpole, S.; Pritchard, A. L.; Cebulla, C. M.; Pilarski, R.; Stautberg, M.; Davidorf, F. H.; de la Fouchardière, A.; Cabaret, O.; Golmard, L.; Stoppa-Lyonnet, D.; Garfield, E.; Njauw, C.-N. N.; Cheung, M.; Turunen, J. A.; Repo, P.; Järvinen, R.-S. S.; Van Doorn, R.; Jager, M. J.; Luyten, G. P. M. M.; Marinkovic, M.; Chau, C.; Potrony, M.; Höiom, V.; Helgadottir, H.; Pastorino, L.; Bruno, W.; Andreotti, V.; Dalmasso, B.; Ciccarese, G.; Queirolo, P.; Mastracci, L.; Wadt, K.; Kiilgaard, J. F.; Speicher, M. R.; van Poppelen, N.; Kilic, E.; Al-Jamal, R. T.; Dianzani, I.; Betti, M.; Bergmann, C.; Santagata, S.; Dahiya, S.; Taibjee, S.; Burke, J.; Poplawski, N.; O’Shea, S. J.; Newton-Bishop, J.; Adlard, J.; Adams, D. J.; Lane, A.-M. M.; Kim, I.; Klebe, S.; Racher, H.; Harbour, J. W.; Nickerson, M. L.; Murali, R.; Palmer, J. M.; Howlie, M.; Symmons, J.; Hamilton, H.; Warrier, S.; Glasson, W.; Johansson, P.; Robles-Espinoza, C. D.; Ossio, R.; de Klein, A.; Puig, S.; Ghiorzo, P.; Nielsen, M.; Kivelä, T. T.; Tsao, H.; Testa, J. R.; Gerami, P.; Stern, M.-H.; Paillerets, B. B.; Abdel-Rahman, M. H.; Hayward, N. K.; Abdel Rahman, M. H.; Kim, I.; Klebe, S.; Racher, H.; Harbour, J. W.; Nickerson, M. L.; Murali, R.; Palmer, J. M.; Howlie, M.; Symmons, J.; Hamilton, H.; Warrier, S.; Glasson, W.; Johansson, P.; Robles-Espinoza, C. D.; Ossio, R.; de Klein, A.; Puig, S.; Ghiorzo, P.; Nielsen, M.; Kivelä, T. T.; Tsao, H.; Testa, J. R.; Gerami, P.; Stern, M.-H.; Paillerets, B. B.; Abdel-Rahman, M. H.; Hayward, N. K. Comprehensive Study of the Clinical Phenotype of Germline BAP1 Variant-Carrying Families Worldwide. *J. Natl. Cancer Inst.* **2018**, *110* (12), 1328–1341. https://doi.org/10.1093/jnci/djy171.

(2) Yu, M. D.; Masoomian, B.; Shields, J. A.; Shields, C. L. BAP1 Germline Mutation Associated with Bilateral Primary Uveal Melanoma. *Ocul. Oncol. Pathol.* **2020**, *6* (1), 10–14. https://doi.org/10.1159/000499570.

(3) Young, K. Z.; Fossum, S. L.; Lowe, L.; Else, T.; Fecher, L. A.; Demirci, H.; Cha, K. B. An Adolescent with Uveal Melanoma and BAP1 Tumor Predisposition Syndrome. *JAAD case reports* **2020**, *6* (6), 563–566. https://doi.org/10.1016/j.jdcr.2020.04.018.

(4) A, Y.; LA, L.; A, M.; CL, S. BAP1 GERMLINE MUTATION WITH IRIDOCILIOCHOROIDAL MELANOMA AND MALIGNANT PERITONEAL MESOTHELIOMA. *Retin. Cases Brief Rep.* **2019**. https://doi.org/10.1097/ICB.0000000000000934.

(5) Chau, C.; Doorn, R. Van; Poppelen, N. M. Van; Stoep, N. Van Der; Ouweland, A. M. W. Van Den; Naus, N. C.; Hout, A. H. Van Der; Potjer, T. P.; Bleeker, F. E.; Wevers, M. R.; van Hest, L. P.; Jongmans, M. C. J.; Marinkovic, M.; Bleeker, J. C.; Jager, M. J.; Luyten, G. P. M.; Nielsen, M. Families with BAP1-Tumor Predisposition Syndrome in The Netherlands : Path to Identification and a Proposal for Genetic Screening Guidelines. *Cancers (Basel).* **2019**, *11*, 1114.

(6) Repo, P.; Järvinen, R. S.; Jäntti, J. E.; Markkinen, S.; Täll, M.; Raivio, V.; Turunen, J. A.; Kivelä, T. T. Population-Based Analysis of BAP1 Germline Variations in Patients with Uveal Melanoma. *Hum. Mol. Genet.* **2019**, *28* (14), 2415–2426. https://doi.org/10.1093/hmg/ddz076.

(7) Pastorino, S.; Yoshikawa, Y.; Pass, H. I.; Emi, M.; Nasu, M.; Pagano, I.; Takinishi, Y.; Yamamoto, R.; Minaai, M.; Hashimoto-Tamaoki, T.; Ohmuraya, M.; Goto, K.; Goparaju, C.; Sarin, K. Y.; Tanji, M.; Bononi, A.; Napolitano, A.; Gaudino, G.; Hesdorffer, M.; Yang, H.; Carbone, M. A Subset of Mesotheliomas with Improved Survival Occurring in Carriers of BAP1 and Other Germline Mutations. *J. Clin. Oncol.* **2018**, *36* (35), 3485–3494. https://doi.org/10.1200/JCO.2018.79.0352.

(8) Yoshikawa, Y.; Emi, M.; Nakano, T.; Gaudino, G. Mesothelioma Developing in Carriers of Inherited Genetic Mutations. *Transl. lung cancer Res.* **2020**, *9* (Suppl 1), S67–S76. https://doi.org/10.21037/tlcr.2019.11.15.

(9) Shinozaki-Ushiku, A.; Kohsaka, S.; Kage, H.; Oda, K.; Miyagawa, K.; Nakajima, J.; Aburatani, H.; Mano, H.; Ushiku, T. Genomic Profiling of Multiple Primary Cancers Including Synchronous Lung Adenocarcinoma and Bilateral Malignant Mesotheliomas: Identification of a Novel BAP1 Germline Variant. *Pathol. Int.* **2020**, *70* (10), 775–780. https://doi.org/10.1111/pin.12977.

(10) Zauderer, M. G.; Jayakumaran, G.; DuBoff, M.; Zhang, L.; Francis, J. H.; Abramson, D. H.; Cercek, A.; Nash, G. M.; Shoushtari, A.; Chapman, P.; D’Angelo, S.; Arnold, A. G.; Siegel, B.; Fleischut, M. H.; Ni, A.; Rimner, A.; Rusch, V. W.; Adusumilli, P. S.; Travis, W.; Sauter, J. L.; Zehir, A.; Mandelker, D.; Ladanyi, M.; Robson, M. Prevalence and Preliminary Validation of Screening Criteria to Identify Carriers of Germline BAP1 Mutations. *J. Thorac. Oncol.* **2019**, *14* (11), 1989–1994. https://doi.org/10.1016/j.jtho.2019.07.002.

(11) Soares De Sá, B. C.; De MacEdo, M. P.; Torrezan, G. T.; Braga, J. C. T.; Fidalgo, F.; Moredo, L. F.; Lellis, R.; Duprat, J. P.; Carraro, D. M.; BC, S. de S.; MP, de M.; GT, T.; JCT, B.; F, F.; LF, M.; R, L.; JP, D.; DM, C. BAP1 Tumor Predisposition Syndrome Case Report: Pathological and Clinical Aspects of BAP1-Inactivated Melanocytic Tumors (BIMTs), Including Dermoscopy and Confocal Microscopy. *BMC Cancer* **2019**, *19* (1), 1–8. https://doi.org/10.1186/s12885-019-6226-8.

(12) Chau, C.; Doorn, R. Van; Poppelen, N. M. Van; Stoep, N. Van Der; Ouweland, A. M. W. Van Den; Naus, N. C.; Hout, A. H. Van Der; Potjer, T. P.; Bleeker, F. E.; Wevers, M. R.; van Hest, L. P.; Jongmans, M. C. J.; Marinkovic, M.; Bleeker, J. C.; Jager, M. J.; Luyten, G. P. M.; Nielsen, M. Families with BAP1-Tumor Predisposition Syndrome in The Netherlands : Path to Identification and a Proposal for Genetic Screening Guidelines. *Cancers (Basel).* **2019**, *11*, 1114.

(13) O’Shea, S. J.; Robles-Espinoza, C. D.; McLellan, L.; Harrigan, J.; Jacq, X.; Hewinson, J.; Iyer, V.; Merchant, W.; Elliott, F.; Harland, M.; Bishop, D. T.; Newton-Bishop, J. A.; Adams, D. J. A Population-Based Analysis of Germline BAP1 Mutations in Melanoma. *Hum. Mol. Genet.* **2017**, *26* (4), 717–728. https://doi.org/10.1093/hmg/ddw403.

(14) Satolli, F.; De Felici Del Giudice, M. B.; Bertolani, M.; Ricci, R.; Lotti, T.; Zucchi, A.; Feliciani, C. BAP1 Tumour Predisposition Syndrome: A New Mutation in One Family. *Acta Derm. Venereol.* **2019**, *99* (11), 1045–1046. https://doi.org/10.2340/00015555-3267.

(15) Wiesner, T.; Obenauf, A. C.; Murali, R.; Fried, I.; Griewank, K. G.; Ulz, P.; Windpassinger, C.; Wackernagel, W.; Loy, S.; Wolf, I.; Viale, A.; Lash, A. E.; Pirun, M.; Socci, N. D.; Rütten, A.; Palmedo, G.; Abramson, D.; Offit, K.; Ott, A.; Becker, J. C.; Cerroni, L.; Kutzner, H.; Bastian, B. C.; Speicher, M. R. Germline Mutations in BAP1 Predispose to Melanocytic Tumors. *Nat. Genet.* **2011**, *43* (10), 1018–1022. https://doi.org/10.1038/ng.910.

(16) Louie, B. H.; Kurzrock, R. *BAP1: Not Just a BRCA1-Associated Protein*; Cancer Treat Rev, 2020; Vol. 90, p 102091. https://doi.org/10.1016/j.ctrv.2020.102091.

(17) Brandi, G.; Deserti, M.; Palloni, A.; Turchetti, D.; Zuntini, R.; Pedica, F.; Frega, G.; De Lorenzo, S.; Abbati, F.; Rizzo, A.; Di Marco, M.; Massari, F.; Tavolari, S. Intrahepatic Cholangiocarcinoma Development in a Patient With a Novel Bap1 Germline Mutation and Low Exposure To Asbestos. *Cancer Genet.* **2020**, No. xxxx, 1–6. https://doi.org/10.1016/j.cancergen.2020.10.001.

(18) Maynard, H.; Stadler, Z. K.; Berger, M. F.; Solit, D. B.; Ly, M.; Lowery, M. A.; Mandelker, D.; Zhang, L.; Jordan, E.; El Dika, I.; Kemel, Y.; Ladanyi, M.; Robson, M. E.; O’Reilly, E. M.; Abou-Alfa, G. K. Germline Alterations in Patients with Biliary Tract Cancers: A Spectrum of Significant and Previously Underappreciated Findings. *Cancer* **2020**, *126* (9), 1995–2002. https://doi.org/10.1002/cncr.32740.

(19) Abdel-Rahman, M. H.; Pilarski, R.; Cebulla, C. M.; Massengill, J. B.; Christopher, B. N.; Boru, G.; Hovland, P.; Davidorf, F. H. Germline BAP1 Mutation Predisposes to Uveal Melanoma, Lung Adenocarcinoma, Meningioma, and Other Cancers. *J. Med. Genet.* **2011**, *48* (12), 856–859. https://doi.org/10.1136/jmedgenet-2011-100156.

(20) Dumbrava, E. I.; Brusco, L.; Daniels, M.; Wathoo, C.; Shaw, K.; Lu, K.; Zheng, X.; Strong, L.; Litton, J.; Arun, B.; Eterovic, A. K.; Routbort, M.; Patel, K.; Qi, Y.; Piha-Paul, S.; Subbiah, V.; Hong, D.; Rodon, J.; Kopetz, S.; Mendelsohn, J.; Mills, G. B.; Chen, K.; Meric-Bernstam, F. Expanded Analysis of Secondary Germline Findings from Matched Tumor/Normal Sequencing Identifies Additional Clinically Significant Mutations. *JCO Precis. Oncol.* **2019**, *3*. https://doi.org/10.1200/PO.18.00143.

(21) Tesch, M. E.; Pater, J. A.; Vandekerkhove, G.; Wang, G.; Binnington, K.; So, A. I.; Wyatt, A. W.; Eigl, B. J. Concurrent Germline and Somatic Pathogenic BAP1 Variants in a Patient with Metastatic Bladder Cancer. *NPJ genomic Med.* **2020**, *5*, 12. https://doi.org/10.1038/s41525-020-0121-8.

(22) Carbone, M.; Pass, H. I.; Ak, G.; Alexander, H. R.; Baas, P.; Baumann, F.; Blakely, A. M.; Bueno, R.; Bzura, A.; Cardillo, G.; Churpek, J. E.; Dianzani, I.; De Rienzo, A.; Emi, M.; Emri, S.; Felley-Bosco, E.; Fennell, D. A.; Flores, R. M.; Grosso, F.; Hayward, N. K.; Hesdorffer, M.; Hoang, C. D.; Johansson, P. A.; Kindler, H. L.; Kittaneh, M.; Krausz, T.; Mansfield, A.; Metintas, M.; Minaai, M.; Mutti, L.; Nielsen, M.; O’Byrne, K.; Opitz, I.; Pastorino, S.; Pentimalli, F.; de Perrot, M.; Pritchard, A.; Ripley, R. T.; Robinson, B.; Rusch, V.; Taioli, E.; Takinishi, Y.; Tanji, M.; Tsao, A. S.; Tuncer, A. M.; Walpole, S.; Wolf, A.; Yang, H.; Yoshikawa, Y.; Zolondick, A.; Schrump, D. S.; Hassan, R. Medical and Surgical Care of Patients With Mesothelioma and Their Relatives Carrying Germline BAP1 Mutations. *J. Thorac. Oncol.* **2022**, *17* (7), 873–889. https://doi.org/10.1016/j.jtho.2022.03.014.
